# Supplementary material for: Cellular metabolism constrains innate immune responses in early human ontogeny
Source: Nat Commun. 2018 Nov 16;9:4822. doi: 10.1038/s41467-018-07215-9 (PMC6240060; doi:10.1038/s41467-018-07215-9)
Supplement: Supplementary file 1 — Supplementary Information [file 41467_2018_7215_MOESM1_ESM.pdf]

SUPPLEMENTARY FIGURES

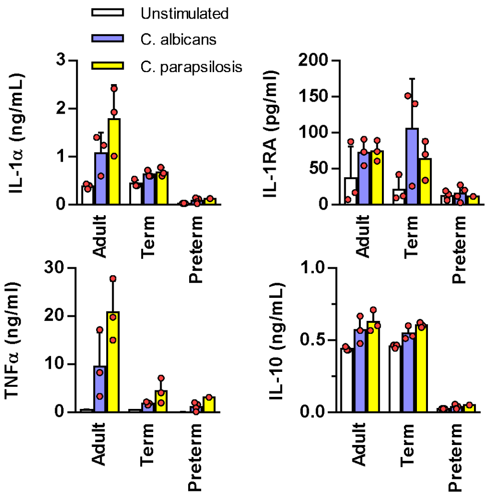

Supplementary Figure 1: Age-related cytokine response to *Candida species.* 24h stimulation of blood mononuclear cells using *Candida albicans* or *parapsilosis* (3 to 4 subjects/age group; preterm samples ranging from 26 to 33 weeks gestation; mean ± SD).

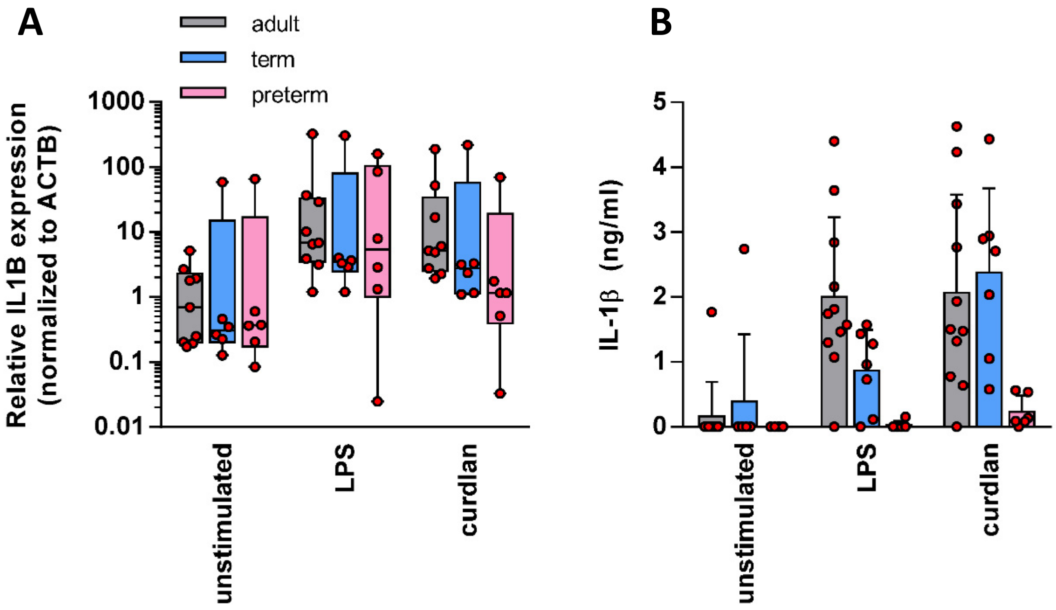

Supplementary Figure 2: Age-related IL-1β transcript (A) and protein (B) expression in response to immune stimulation. Data from 6 to 11 subjects per age group; left panel: box and whiskers; right panel: bars = mean ± SD.

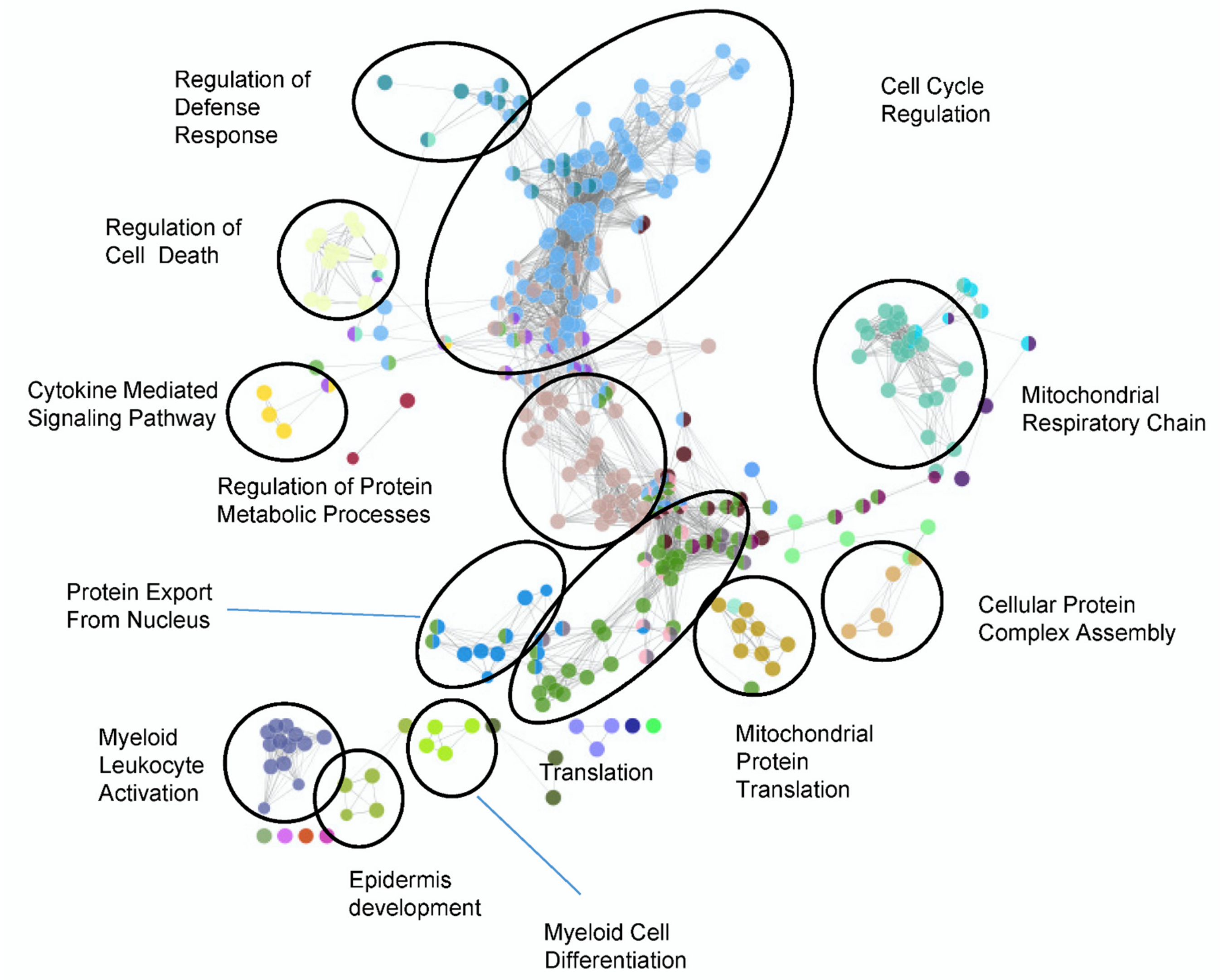

Supplementary Figure 3: Gene-set enrichment analysis of clusters (colors, with pathway names) of differentially expressed genes (FDR<0.01) across age groups (unstimulated preterm, term and adult monocytes; data from same samples as in figure 3, 4A-C and 7H).

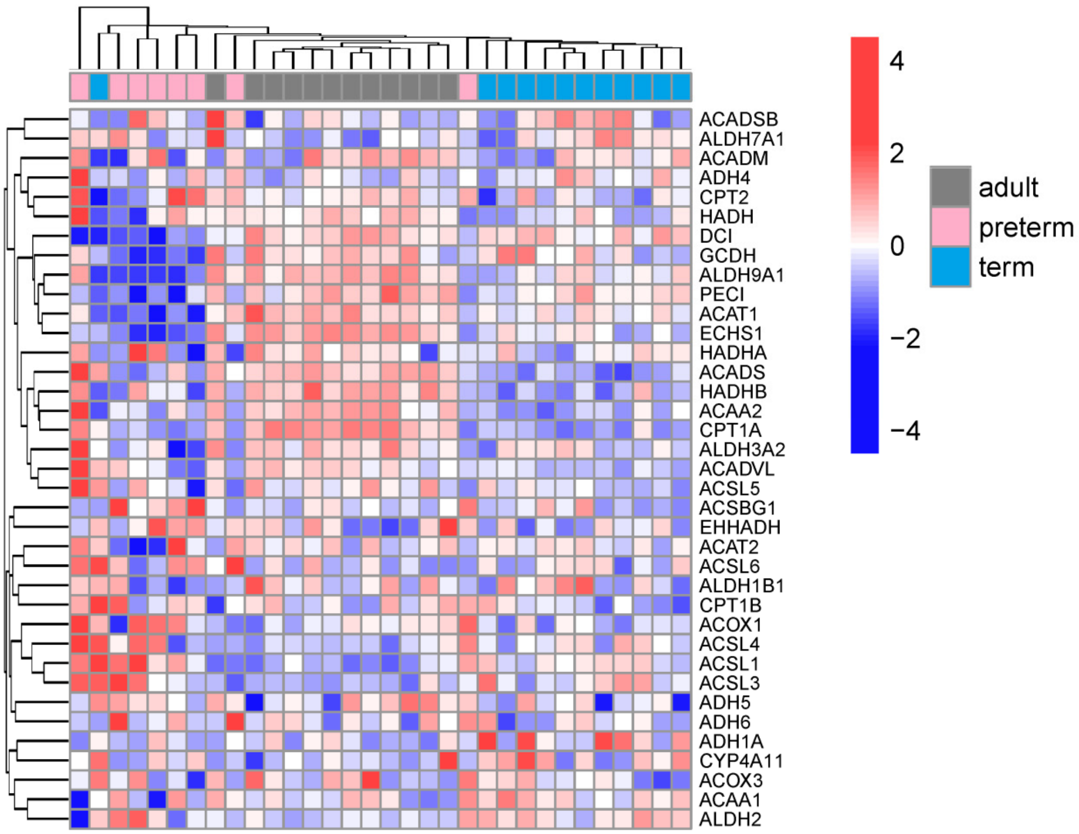

Supplementary Figure 4: Age-related changes in expression of genes involved in fatty acid degradation (unstimulated monocytes). Unsupervised clustering; Scale represents z-score; Data from same samples as in figure 3, 4A-C and 7H.

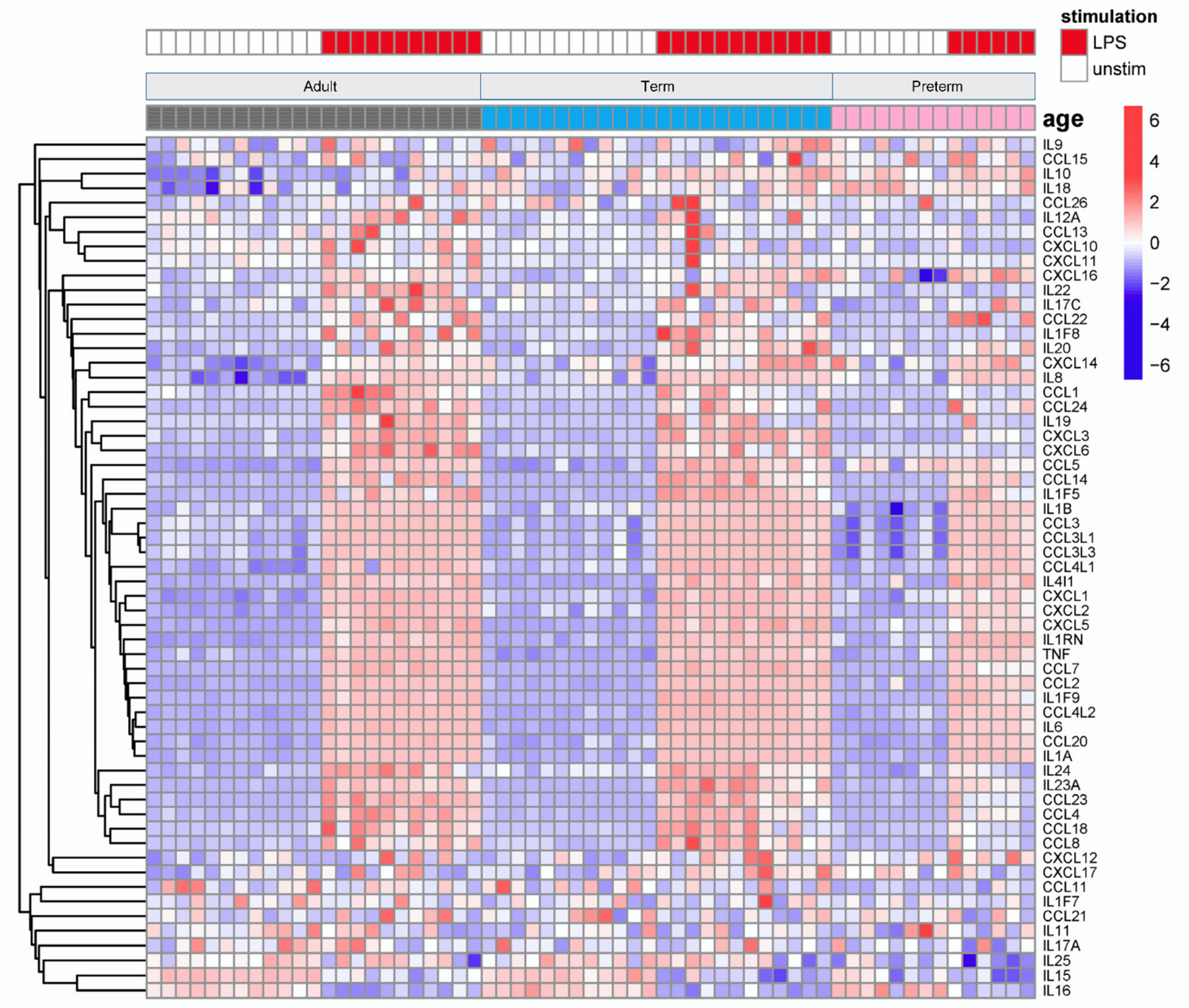

Supplementary Figure 5: Age-related changes in cytokine/chemokine gene expression following LPS-stimulation (monocytes); Scale represents z-score; data from same samples used in figure 3, 4A-C and 7H.

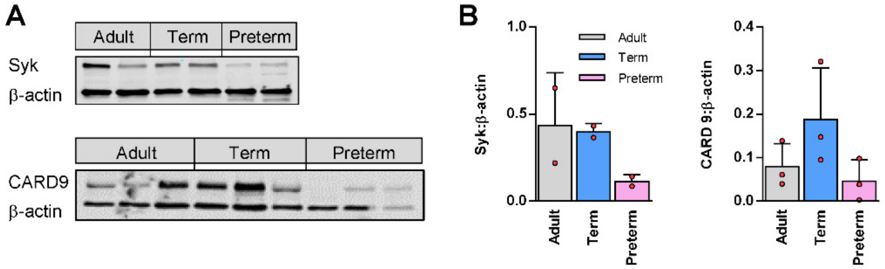

Supplementary Figure 6: Age-related expression Syk and Card9. (A) Representative Western blot (unstimulated monocytes) including a 27 (Syk) and 28 weeks (CARD9) gestation preterm sample**.** Images cropped from same blot probes with each antibody; **(B)** Cumulative quantification from 2 (for Syk) and 3 (for CARD9) independent experiments (mean ± SD).

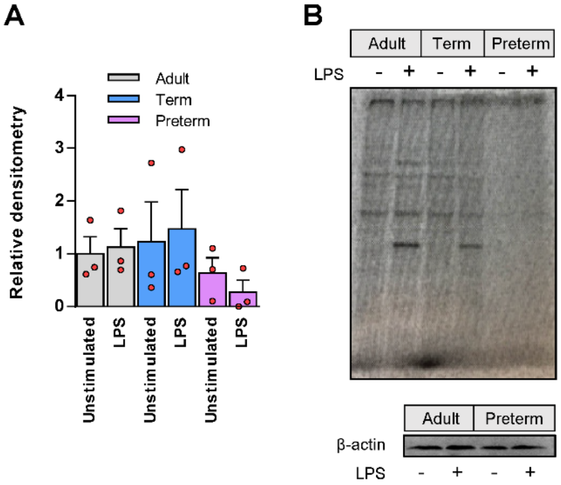

Supplementary Figure 7: Age-related translation response in neonatal monocytes. (A) Cumulative data from three independent ^{35}S-met/cys pulse labelling experiments (preterm samples between 25 and 29 weeks'; mean ± SD); **(B)** representative pulse labelling with Western blotting of same adult/preterm (29 weeks gestation) cell lysates ran separately and probed for β-actin (there was insufficient residual sample from the term subjects for simultaneous β-actin blotting). Equal loading of protein in each lane was confirmed by Bradford method. Densitometry data in **(A)** were measured from each (entire) lane. Monocyte viability was confirmed (>95%) immediately before each experiment, by trypan blue staining.

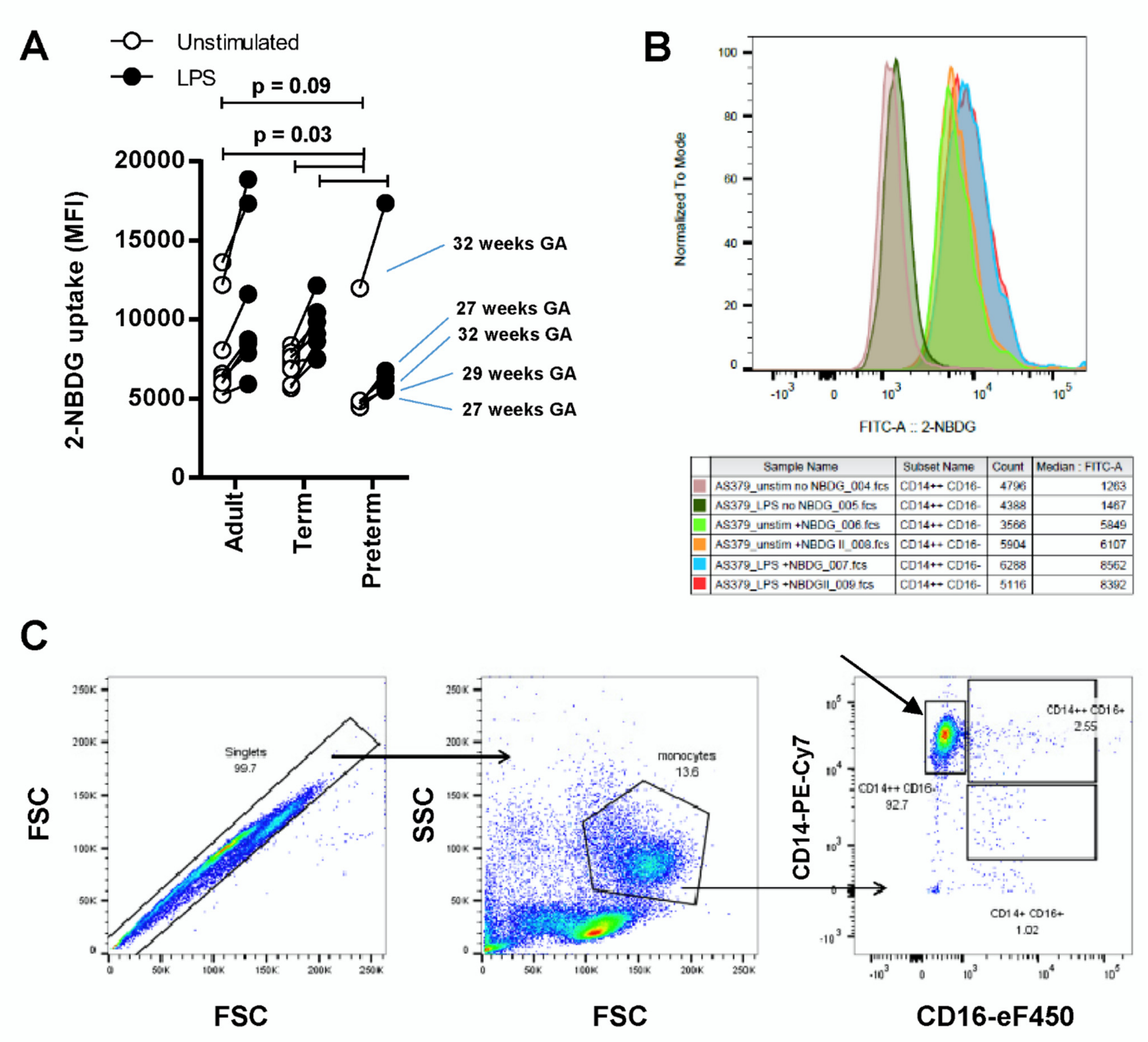

Supplementary Figure 8: Age-related glucose uptake (monocytes). (A) Fluorescent 2-(N-[7-Nitrobenz-2-oxa-1,3-diazol-4-yl]Amino)-2-Deoxyglucose (2NBDG) uptake measured by flow cytometry comparing preterm, term and adult monocytes, at rest and following LPS stimulation (n = 7 adult, 7 term and 5 preterm samples). P value represents difference between groups using a Kolmogorov-Smirnov test (for non-parametric data). Gestational age labels for preterm samples are rounded to the closest week; **(B)** Representative flow cytometry histogram and **(C)** gating strategy for 2-NBDG measures on CD14-high (++)/CD16-negative expressing cells, from an adult sample.

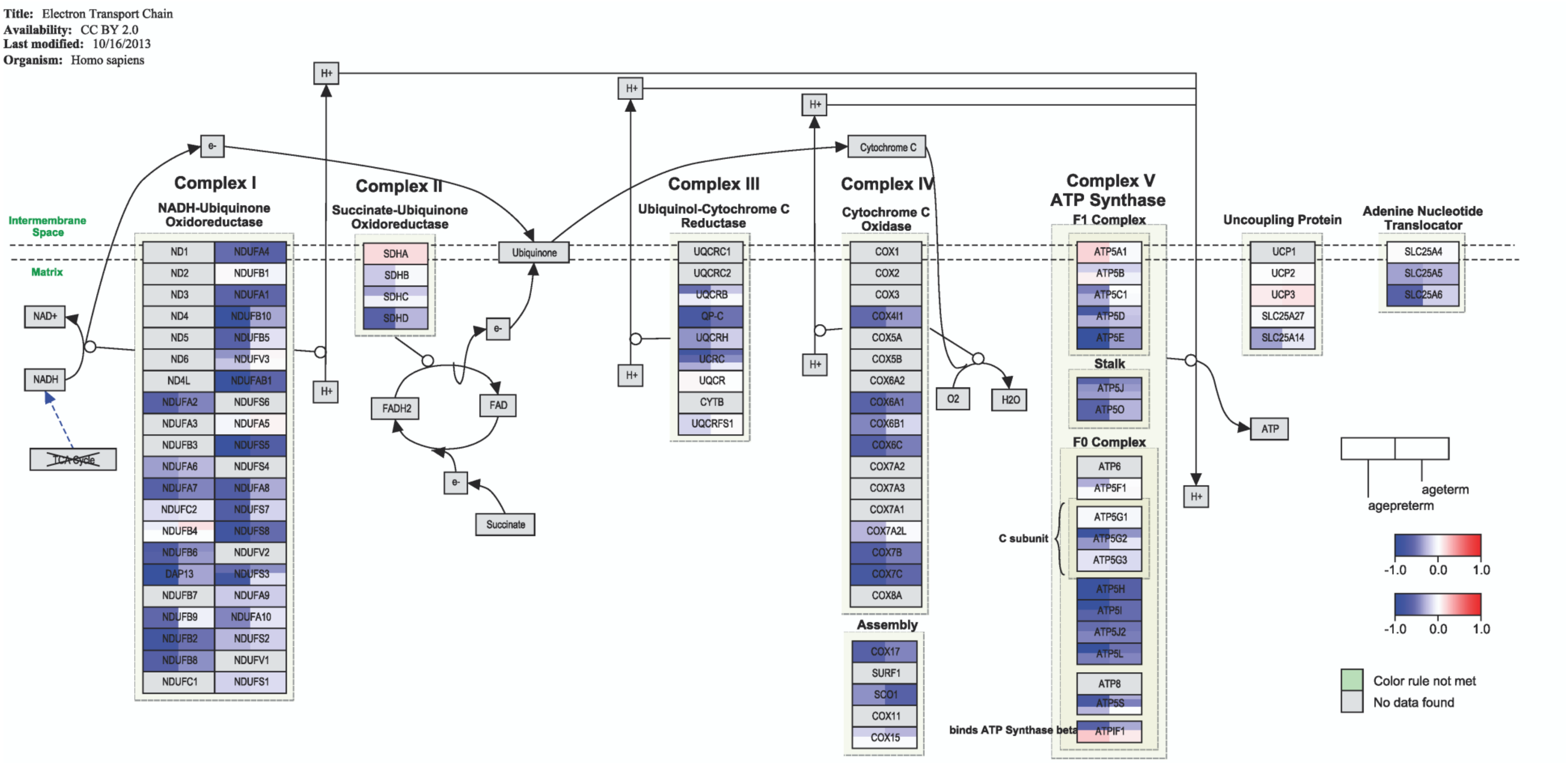

Supplementary Figure 9: Age-related changes in expression of genes involved in mitochondrial electron-transport. Depiction of differential expression in genes involved in Electron Transport Chain. Data from same samples used in figure 3, 4A-C and 7H. Image was created using the PathVisio software at: https://www.pathvisio.org/about/cite-us/. Log fold change of genes significantly affect by age (Limma, unstimulated and LPS samples, FDR 5%) is depicted comparing preterm (left of boxes) and term (right of boxes) samples to adults. Blue/red indicate decreased/increased expression in newborns compared to adults. Scale represents expression changes (log 2).

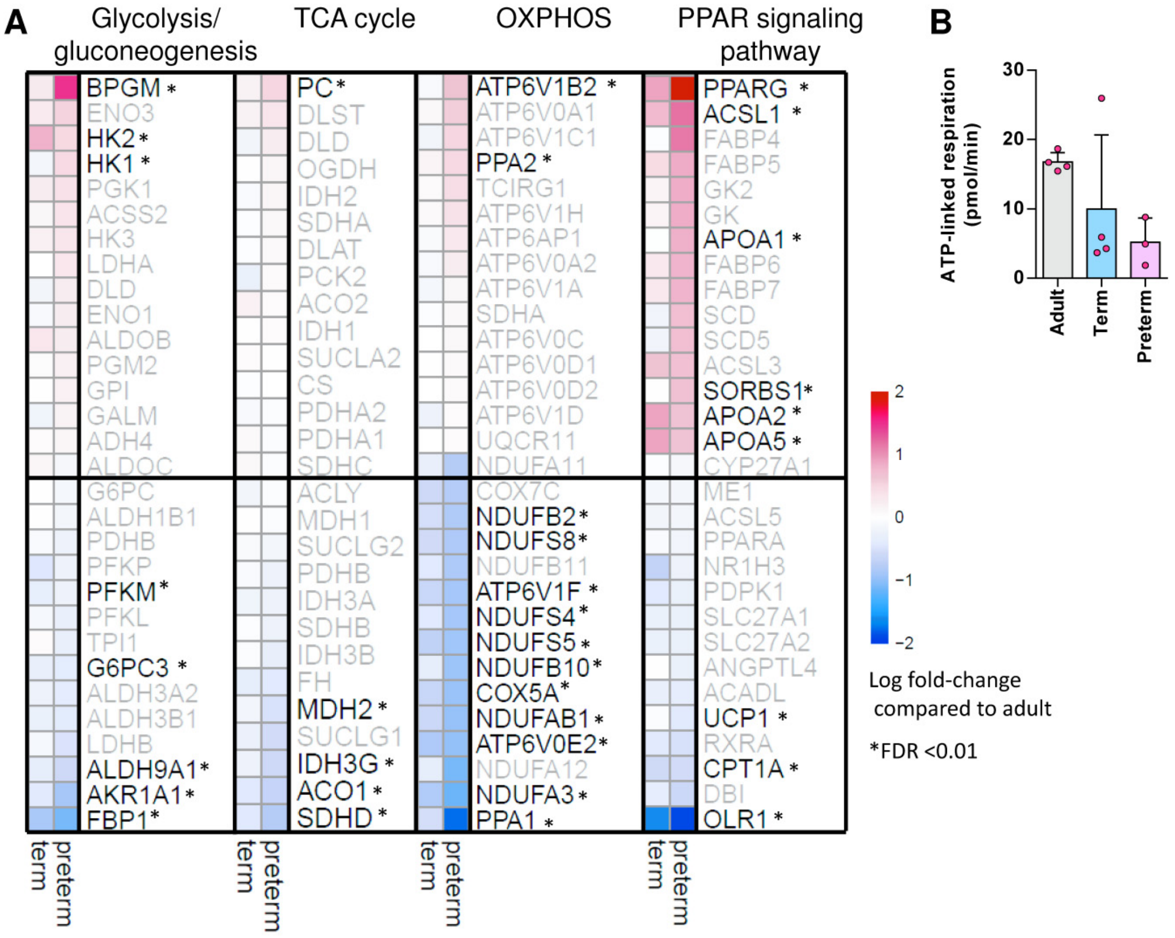

Supplementary Figure 10: Energetic re-wiring in preterm monocytes. (A) Heatmap of top 15 age-dependent up- and down-regulated genes (log fold-change) within each energetic pathway. Genes with a statistically significant differential expression (linear modelling; FDR<1%) between the age groups (all samples, unstimulated and LPS) are marked by an asterisk. Data from same samples used in figure 3, 4A-C and 7H**; (B)** Differential oxygen consumption rate after addition of oligomycin in glucose-containing culture media, in monocytes (bars: mean ± SD), 3 – 4 subjects per age group.

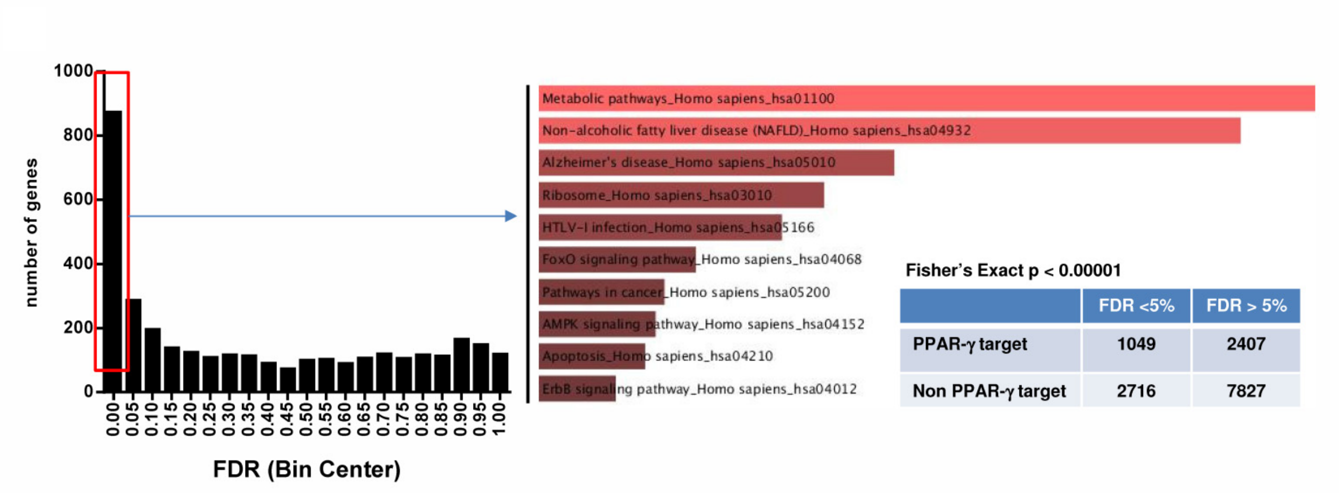

Supplementary Figure 11: Increased PPAR-γ activity in preterm monocytes is suggested by a significant enrichment of its target genes and mapping of these genes to metabolic pathways. Linear modeling was performed on all samples (both unstimulated and LPS-stimulated isolated monocytes) to identify differentially expressed genes by "age". Differentially expressed genes (FDR 5%) were then compared to known PPAR-γ gene targets from the ChEA database (Lachman A et al, Bioinformatics 2010). Significant enrichment of PPAR-γ targets was tested by Fisher's Exact test followed by gene set enrichment analyses of differentially expressed PPAR-γ gene targets using Enrichr[1]. Data from same samples used in figure 3, 4A-C and 7H

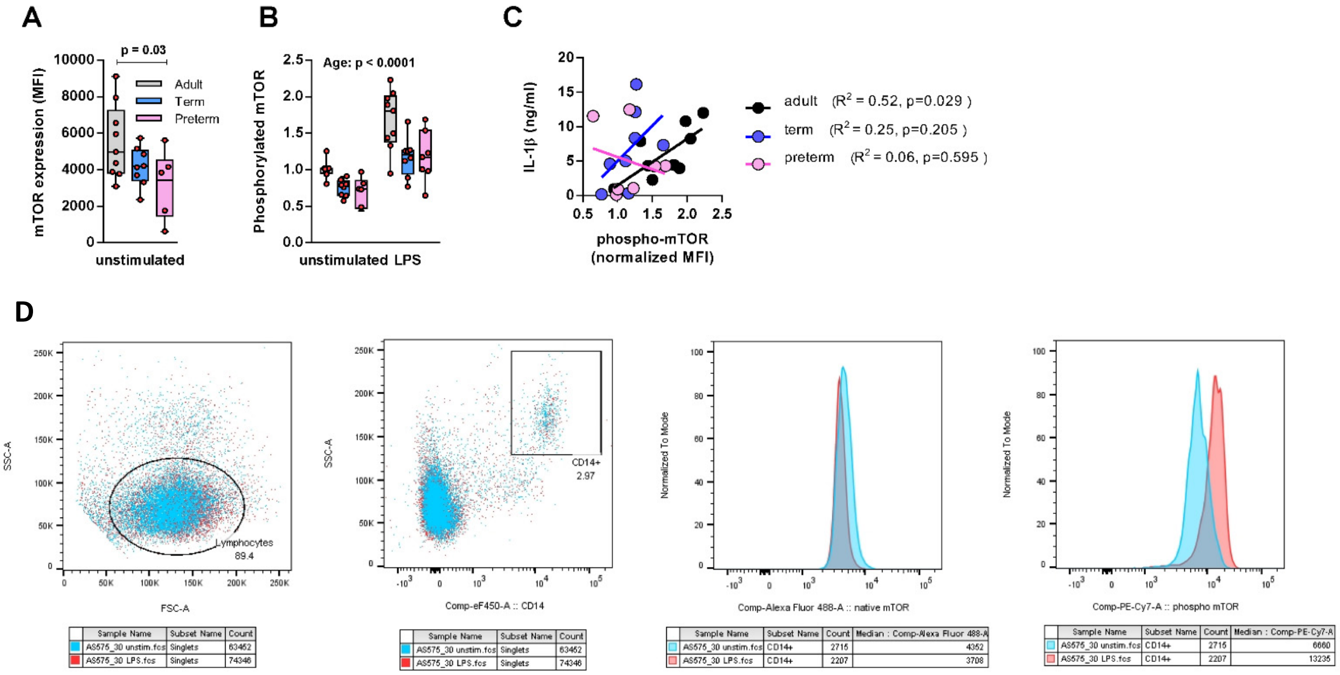

Supplementary Figure 12: **(A)** mTOR expression (2-sided unpaired t-test) and **(B)** phosphorylation after LPS stimulation (30 min; age-related effect by 2-way ANOVA), by flow cytometry (boxes and whiskers). 7 to 8 subjects per age group, except for some preterm samples had insufficient cells for an unstimulated condition; **(C)** Linear regression between LPS-induced mTOR phosphorylation and cytokine production (with goodness of fit R^2 and p value). **(D)** Exemplary gating for native mTOR protein and mTOR phosphorylation detection. Median fluorescence intensity of mTOR phosphorylation normalized on unstimulated cells.

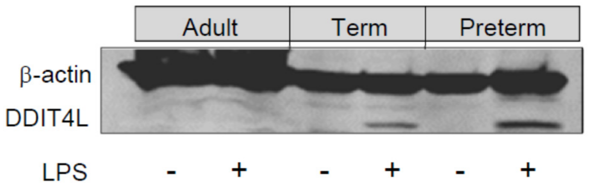

Supplementary Figure 13: Increased expression of DDIT4L protein in preterm monocytes (cells are from a preterm subject at 27 weeks gestation) analyzed by Western blot after 2.5 hours of LPS stimulation. Images cropped from same blot probes with each antibody.

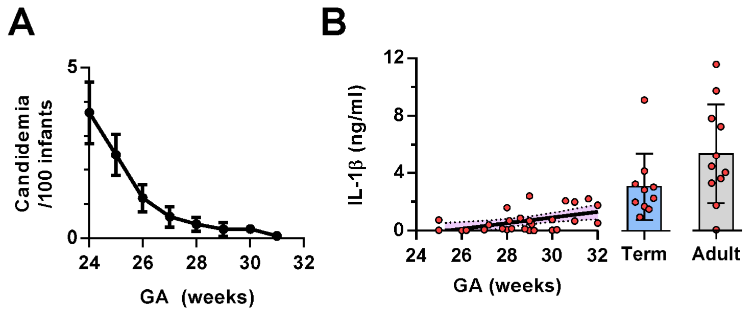

Supplementary Figure 14: (A) Mean gestational age-related prevalence of Candidemia in infants born below 33 weeks of gestation in Canada between 2003 and 2013 (39,336 infants; bars = 95% CI). Data was provided by the Canadian Neonatal Network (www.canadianneonatalnetwork.org); **(B)** Candida-induced production of IL-1β by gestational age in preterm (GA), or term neonatal (n=12) and adult controls (n=18; mean ± SD); pink area and error bars = 95%CI.

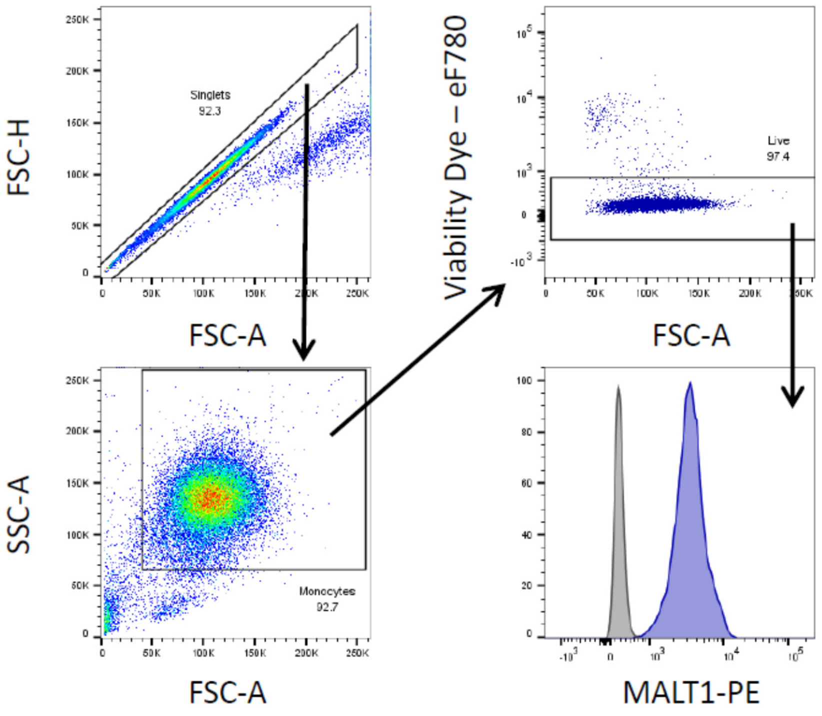

Supplementary Figure 15: Gating strategy for MALT1 protein detection by flow cytometry, including a histogram (bottom right) of MALT1 detection (blue) and fluorescence-minus MALT1 staining control (grey).

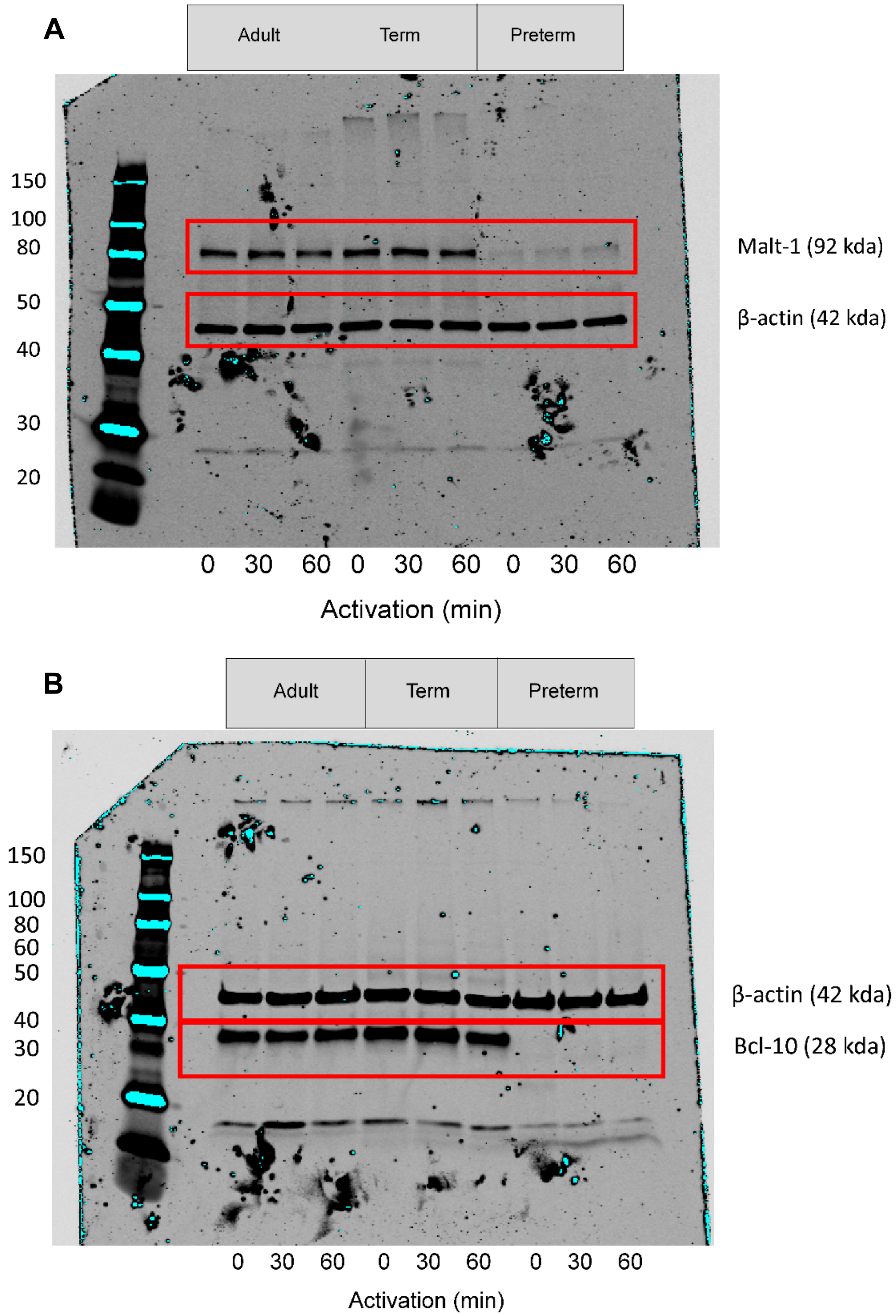

Supplementary Figure 16: Uncropped Western blot image for figure 5F **(A)** MALT and **(B)** Bcl10.

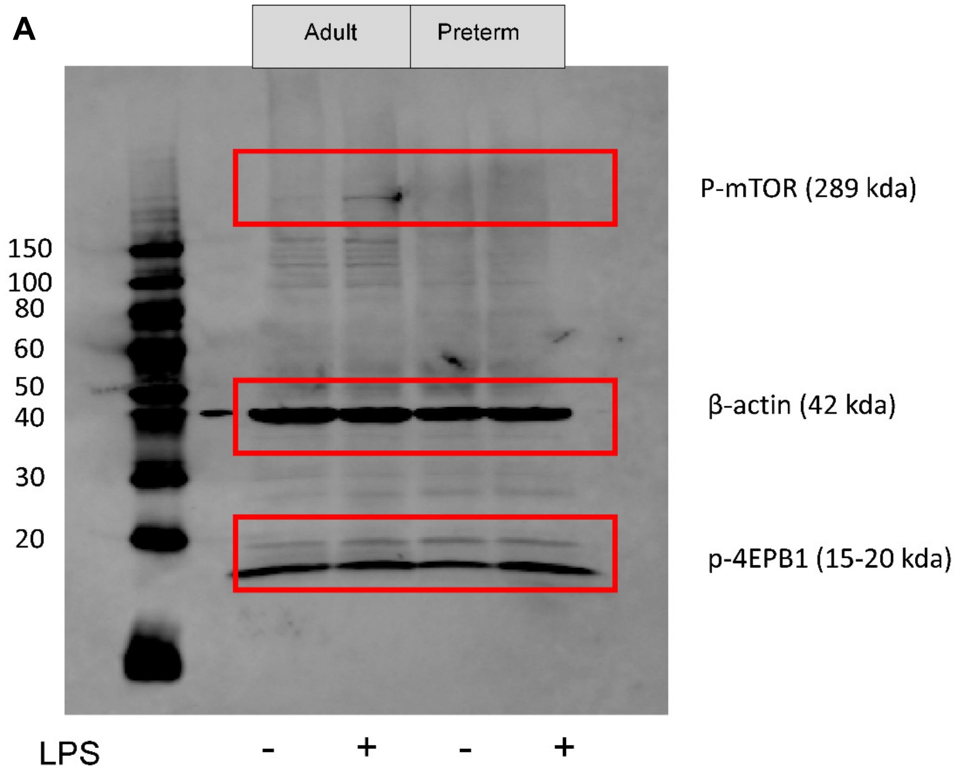

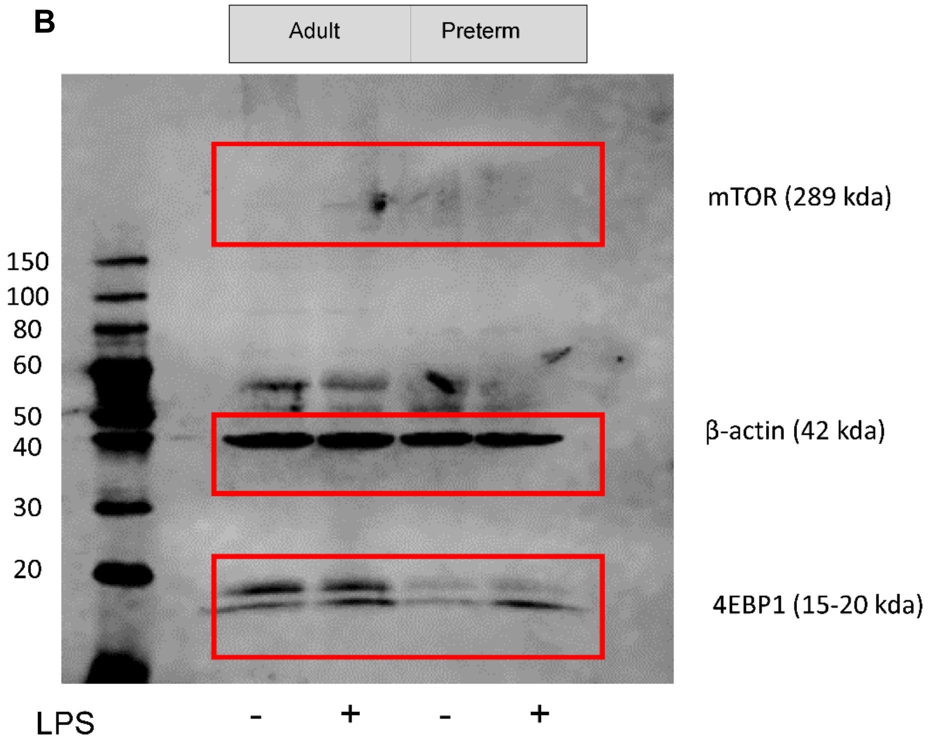

Supplementary Figure 17: Uncropped Western blot image for figure 7G **(A)** Phosphorylated mTOR (P-mTOR) and 4EBP1 and **(B)** mTOR and 4EBP1.

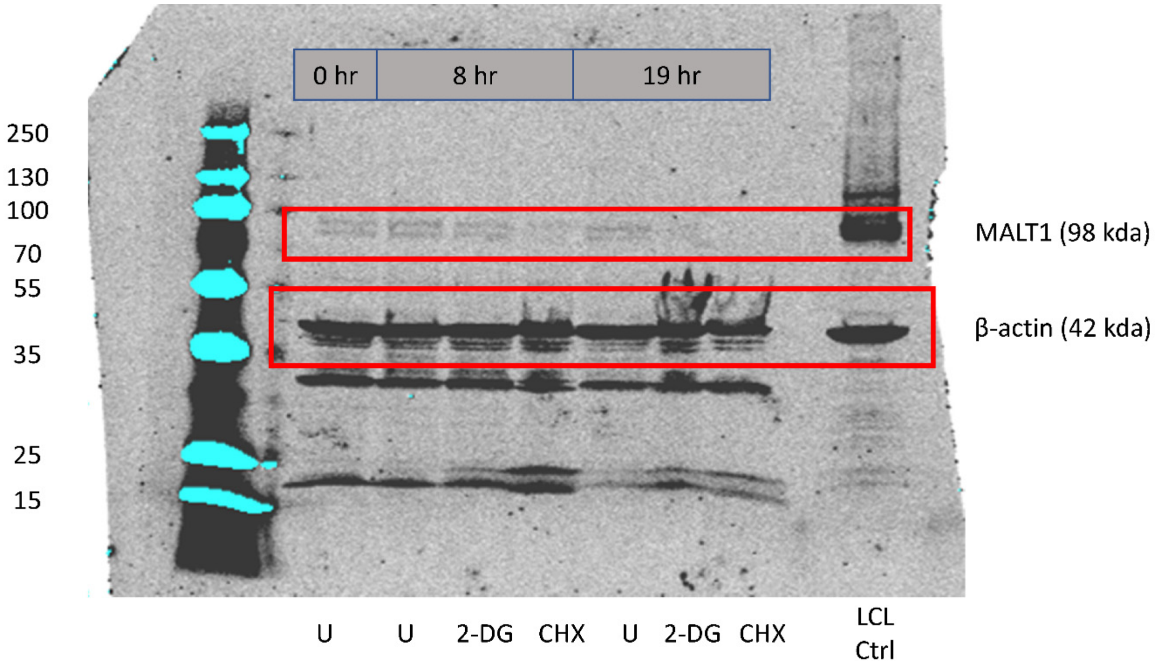

Supplementary Figure 18: Uncropped Western blot image for figure 8A. U = Unstimulated, 2-DG and CHX = cycloheximide conditions. LCL = Lymphoblastoid cell line (positive control for MALT1 expression).

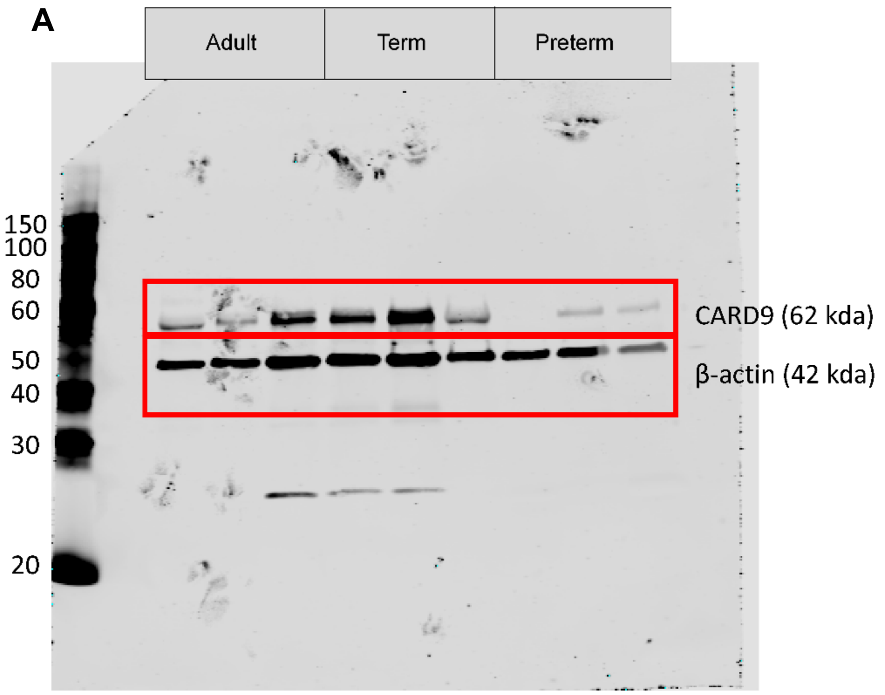

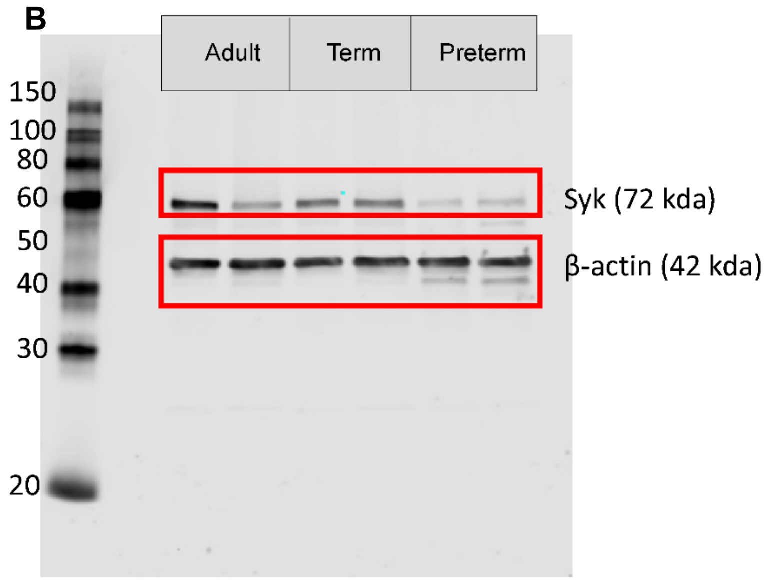

Supplementary Figure 19: Uncropped Western blot image for Supplementary figure 6A; **(A)** CARD9 and **(B)** Syk.

SUPPLEMENTARY TABLES

	Gestational age (weeks)							
	24	25	26	27	28	29	30	31 and 32
Total number of infants	1822	2618	2975	3493	4124	4492	5560	14252
Total number of infants who had infection(s)	702	981	861	867	772	655	521	714
Total infants with *Candida* infections	67	64	35	22	17	12	15	10

Supplementary table 1: Gestational age-related prevalence of infections (all types of micro-organisms) in infants born below 33 weeks of gestation in Canada between 2003 and 2013. Data was provided by the Canadian Neonatal Network (www.canadianneonatalnetwork.org).

*For all tables below, indicate missing clinical data either because the data could not be found, or because access to medical chart was declined by parents/legal guardians.

	**Figure panel**					
**Clinical variable**	**A**	**B, C**	**D**	**E**	**F**	**G**
N =	16	10	21	See figure legend	23	12
Gestational age, mean ± SD (weeks)	29.1 ± 2.6	30.0 ± 2.4	28.7 ± 2.0		28.2 ± 2.2	29.8 ± 2.3
Birth weight, mean ± SD (weeks)	1278 ± 443	1397 ± 508	1174 ± 407		1111 ± 308	1357 ± 511
Sex, % male	40	60	50		52	64
Received antenatal corticosteroids, % yes	94	80	79		91	83
Labour, % yes	75	60	55		64	58
Received magnesium sulfate, % yes	63	50	70		73	42
Mode of delivery, % C-section	50	30	45		55	42
N = with at least one missing value*	1	0	2		2	1

Supplementary Table 2: Additional clinical data for preterm subjects in figure 1**.**

	Figure panel		
**Clinical variable**	**A**	**B**	**C, D**
N = *	N/A	16	4
Gestational age, mean ± SD (weeks)		29.1 ± 2.6	27.6 ± 1.9
Birth weight, mean ± SD (weeks)		1259 ± 481	1162 ± 350
Sex, % male		43	50
Received antenatal corticosteroids, % yes		92	100
Labour, % yes		67	100
Received magnesium sulfate, % yes		60	100
Mode of delivery, % C-section		53	50
N = with at least one missing value*		2	0

Supplementary Table 3: Additional clinical for preterm subjects in figure 2.

Clinical variable	
N = *	8
Gestational age, mean ± SD (weeks)	26.9 ± 1.6
Birth weight, mean ± SD (weeks)	1038 ± 205
Sex, % male	50
Received antenatal corticosteroids, % yes	100
Labour, % yes	75
Received magnesium sulfate, % yes	63
Mode of delivery, % C-section	38
N = with at least one missing value*	0

Supplementary Table 4: Additional clinical for preterm subjects in figure 3, 4A, B and C, and Supplementary Figures 3, 4, 5, 9, 10A and 11.

Clinical variable	
N = *	7
Gestational age, mean ± SD (weeks)	29.7 ± 3.2
Birth weight, mean ± SD (weeks)	1544 ± 550
Sex, % male	80
Received antenatal corticosteroids, % yes	60
Labour, % yes	60
Received magnesium sulfate, % yes	80
Mode of delivery, % C-section	60
N = with at least one missing value*	2

Supplementary Table 5: Additional clinical for preterm subjects in figure 4D (includes 4 samples in Supplementary Figure 1).

	Figure panel					
**Clinical variable**	**A**	**B, C**	**D**	**E**	**F**	**G, H**
N = *	N/A	4	6	10	See figure legend	4
Gestational age, mean ± SD (weeks)		28.8 ± 1.4	27.6 ± 2.1	27.6 ± 2.3		29.6 ± 1.1
Birth weight, mean ± SD (weeks)		1457 ± 215	1039 ± 209	991 ± 324		1314 ± 205
Sex, % male		100	50	38		50
Received antenatal corticosteroids, % yes		100	100	89		100
Labour, % yes		50	83	56		75
Received magnesium sulfate, % yes		75	50	78		50
Mode of delivery, % C-section		75	17	56		50
N = with at least one missing value*		0	0	1		0

Supplementary Table 6: Additional clinical for preterm subjects in figure 5.

	Figure panel					
**Clinical variable**	**A**	**C**	**D**	**E,F**	**G**	**H**
N = *	N/A	3	10	N/A	See figure legend	Same as Figure 3, 4A, B, C
Gestational age, mean ± SD (weeks)		28.8 ± 2.3	28.8 ± 3.1			
Birth weight, mean ± SD (weeks)		1330 ± 625	1220 ± 575			
Sex, % male		0	56			
Received antenatal corticosteroids, % yes		100	100			
Labour, % yes		33	60			
Received magnesium sulfate, % yes		33	70			
Mode of delivery, % C-section		67	60			
N = with at least one missing value*		0	1			

Supplementary Table 7: Additional clinical for preterm subjects in figure 7 and Supplementary Figure 10B (data for figure panel 7C).

**Clinical variable**	
N = *	4
Gestational age, mean ± SD (weeks)	30.4 ± 3.3
Birth weight, mean ± SD (weeks)	1668 ± 550
Sex, % male	100
Received antenatal corticosteroids, % yes	50
Labour, % yes	50
Received magnesium sulfate, % yes	75
Mode of delivery, % C-section	50
N = with at least one missing value*	0

Supplementary Table 8: Additional clinical for preterm subjects in Supplementary figure 1.

Clinical variable	A, B
N = *	6
Gestational age, mean ± SD (weeks)	26.8 ± 1.7
Birth weight, mean ± SD (weeks)	1066.3 ± 281
Sex, % male	67
Received antenatal corticosteroids, % yes	100
Labour, % yes	83
Received magnesium sulfate, % yes	67
Mode of delivery, % C-section	67
N = with at least one missing value*	0

Supplementary Table 9: Additional clinical for preterm subjects in Supplementary figure 2.

Clinical variable	
N = *	3
Gestational age, mean ± SD (weeks)	29.0 ± 2.6
Birth weight, mean ± SD (weeks)	940.3 ± 177
Sex, % male	67
Received antenatal corticosteroids, % yes	67
Labour, % yes	33
Received magnesium sulfate, % yes	67
Mode of delivery, % C-section	100
N = with at least one missing value*	1

Supplementary Table 10: Additional clinical for preterm subjects in Supplementary figure 6B.

Clinical variable	
N = *	6
Gestational age, mean ± SD (weeks)	26.7 ± 1.3
Birth weight, mean ± SD (weeks)	979 ± 490
Sex, % male	20
Received antenatal corticosteroids, % yes	80
Labour, % yes	60
Received magnesium sulfate, % yes	80
Mode of delivery, % C-section	100
N = with at least one missing value*	1

Supplementary Table 11: Additional clinical for preterm subjects in Supplementary Figure 7A (including 2 pooled samples in 1st experiment and 3 pooled samples in 3rd experiment).

Clinical variable	
N = *	5
Gestational age, mean ± SD (weeks)	28.9 ± 2.2
Birth weight, mean ± SD (weeks)	1211 ± 691
Sex, % male	25
Received antenatal corticosteroids, % yes	100
Labour, % yes	25
Received magnesium sulfate, % yes	100
Mode of delivery, % C-section	75
N = with at least one missing value*	1

Supplementary Table 12: Additional clinical for preterm subjects in Supplementary Figure 8A.

Clinical variable	
N = *	7
Gestational age, mean ± SD (weeks)	29.0 ± 2.7
Birth weight, mean ± SD (weeks)	1311 ± 609
Sex, % male	86
Received antenatal corticosteroids, % yes	86
Labour, % yes	71
Received magnesium sulfate, % yes	43
Mode of delivery, % C-section	71
N = with at least one missing value*	0

Supplementary Table 13: Additional clinical for preterm subjects in Supplementary Figure 12A-C.

Clinical variable	
N = *	20
Gestational age, mean ± SD (weeks)	28.8 ± 2.1
Birth weight, mean ± SD (weeks)	1181 ± 417
Sex, % male	47
Received antenatal corticosteroids, % yes	79
Labour, % yes	53
Received magnesium sulfate, % yes	74
Mode of delivery, % C-section	47
N = with at least one missing value*	1

Supplementary Table 14: Additional clinical for preterm subjects in Supplementary Figure 14B.

Gene/primer name	Direction	Sequence
Clec7a (dectin-1 receptor)	forward	5'- TCGACTCTCAAAGCAATACCAG -3'
	reverse	5'- CCACAGCTATCACCAGTATTACC -3'
Malt1	forward	5'-AGGCTATGGAACACACTGAAG-3'
	reverse	5'-ACCACTGATATTGAACAAAAGGATG-3'
Card9	forward	5'-CACCCAGCTCTCAGACAAAG-3'
	reverse	5'-CTTAACAAACGGCCCCAATG-3'
Bcl10	forward	5'-GAAATATAAAACTAGAACATCTGAAAGGAC-3'
	reverse	5'-TGGTACATGACAGTGGATGC-3'
Actb	forward	5'-CCTTGCACATGCCGGAG-3'
	reverse	5'-ACAGAGCCTCGCCTTTG-3'
dsRED	forward	5'- TGAAGCTGAAGGTGACCAAG-3'
	reverse	5'- GGACAGCTTCTTGTAGTCG-3'

Supplementary table 15: Primer sequence used in qPCR gene amplification.

SUPPLEMENTARY REFERENCE

1. Kuleshov, M.V., *et al.* Enrichr: a comprehensive gene set enrichment analysis web server 2016 update. *Nucleic Acids Res* **44**, W90-97 (2016).
