# Supplementary material for: Cellular metabolism constrains innate immune responses in early human ontogeny
Source: Nat Commun. 2018 Nov 16;9:4822. doi: 10.1038/s41467-018-07215-9 (PMC6240060; doi:10.1038/s41467-018-07215-9)
Supplement: Supplementary file 3 — Description of Additional Supplementary Files [file 41467_2018_7215_MOESM3_ESM.pdf]

Description of Additional Supplementary Files

Supplementary Data 1

Description: Gene Ontology analysis based on network of differentially expressed genes (adjusted p<0.05) in unstimulated preterm monocytes compared to the combined groups of term and adult monocytes. Cluster 1 includes upregulated genes, whereas cluster 2 includes downregulated genes (selected for input into the analysis based on >2-fold differential expression; FDR<0.01). Sorted by decreasing number of genes in cluster 2.

Supplementary Data 2

Description: Gene Ontology analysis based on network of genes significantly differentially expressed (adjusted p<0.05) between LPS stimulated and unstimulated preterm monocytes, compared to the combined groups of term and adult monocytes. Cluster 1 includes genes upregulated and cluster 2 included genes downregulated upon LPS (selected for input into the analysis based on >2-fold differential expression; FDR<0.01). Sorted by decreasing adjusted significance.

Supplementary Data 3

Description: Normalized expression values (log2 scale) of manually selected genes involved in the mTOR pathway.
