# Supplementary material for: Cellular metabolism constrains innate immune responses in early human ontogeny
Source: Nat Commun. 2018 Nov 16;9:4822. doi: 10.1038/s41467-018-07215-9 (PMC6240060; doi:10.1038/s41467-018-07215-9)
Supplement: Supplementary file 7 — Reporting Summary [file 41467_2018_7215_MOESM7_ESM.pdf]

nature research

Corresponding author(s): Pascal Lavoie

Reporting Summary

Nature Research wishes to improve the reproducibility of the work that we publish. This form provides structure for consistency and transparency in reporting. For further information on Nature Research policies, see Authors & Referees and the Editorial Policy Checklist.

Statistical parameters

When statistical analyses are reported, confirm that the following items are present in the relevant location (e.g. figure legend, table legend, main text, or Methods section).

n/a	Confirmed	
☐	☒	The exact sample size (n) for each experimental group/condition, given as a discrete number and unit of measurement
☐	☒	An indication of whether measurements were taken from distinct samples or whether the same sample was measured repeatedly
☐	☒	The statistical test(s) used AND whether they are one- or two-sided<br>*Only common tests should be described solely by name; describe more complex techniques in the Methods section.*
☐	☒	A description of all covariates tested
☐	☒	A description of any assumptions or corrections, such as tests of normality and adjustment for multiple comparisons
☐	☒	A full description of the statistics including central tendency (e.g. means) or other basic estimates (e.g. regression coefficient) AND variation (e.g. standard deviation) or associated estimates of uncertainty (e.g. confidence intervals)
☒	☐	For null hypothesis testing, the test statistic (e.g. F, t, r) with confidence intervals, effect sizes, degrees of freedom and P value noted<br>*Give P values as exact values whenever suitable.*
☒	☐	For Bayesian analysis, information on the choice of priors and Markov chain Monte Carlo settings
☒	☐	For hierarchical and complex designs, identification of the appropriate level for tests and full reporting of outcomes
☐	☒	Estimates of effect sizes (e.g. Cohen's d, Pearson's r), indicating how they were calculated
☐	☒	Clearly defined error bars<br>*State explicitly what error bars represent (e.g. SD, SE, CI)*

Our web collection on statistics for biologists may be useful.

Software and code

Policy information about availability of computer code

Data collection	The data collected was entered either directly in GraphPad or in an excel spreadsheet.
Data analysis	GraphPad Prism v 6.07 was used for statistical analyses (as indicated in Statical analysis section of the methods). Microscopy images (Figure 1A) were collected and analyzed using IDEAS. Flow cytometry data was analyzed using FlowJo.

For manuscripts utilizing custom algorithms or software that are central to the research but not yet described in published literature, software must be made available to editors/reviewers upon request. We strongly encourage code deposition in a community repository (e.g. GitHub). See the Nature Research guidelines for submitting code & software for further information.

Data

Policy information about availability of data

All manuscripts must include a data availability statement. This statement should provide the following information, where applicable:

- Accession codes, unique identifiers, or web links for publicly available datasets
- A list of figures that have associated raw data
- A description of any restrictions on data availability

Data availability statement has been included: Microarray data used in Fig. 3, 4A-C, 7H, Supplementary Table 1, 2 and 3, and Supplementary Fig. 3, 4, 5, 9 10A and 11 have been deposited in GEO under accession code GSE104510 [https://www.ncbi.nlm.nih.gov/geo/query/acc.cgi?acc=GSE104510]. Uncut Western blot figures

are provided as Supplementary Information. All other data, including raw data used in each figure will be provided upon reasonable request to the corresponding author, and provided that the nature of the request complies with our institutional ethics board policy.

Field-specific reporting

Please select the best fit for your research. If you are not sure, read the appropriate sections before making your selection.

☒ Life sciences ☐ Behavioural & social sciences ☐ Ecological, evolutionary & environmental sciences

For a reference copy of the document with all sections, see nature.com/authors/policies/ReportingSummary-flat.pdf

Life sciences study design

All studies must disclose on these points even when the disclosure is negative.

Sample size	For each experiment, sample size was estimated based on the variance from initial 3 to 5 replicates per preterm, term or adult age group.
Data exclusions	Experiments were routinely conducted to optimize each of the experimental conditions in this article, often using adult or term neonatal samples. These experiments are not included in the article. Except for optimization experiments, no data were excluded.
Replication	Experiments were repeated at least twice. The number of replicate experiments have been indicated throughout the article.
Randomization	Human subjects were randomly recruited over time, to balance the number of replicates between 3 age groups: preterms, terms and adults. Over the course of the study, some efforts were made to include preterm subjects of specific gestational age for a specific experiment, such that the gestational age distributions of the preterm samples were comparable from one experiment to another.
Blinding	Investigators were not blind to the allocation of samples between age group. This was not possible given that the samples came from easily identifiable donor sources.

Reporting for specific materials, systems and methods

Materials & experimental systems

n/a	Involved in the study	
☒	☐	Unique biological materials
☒	☐	Antibodies
☒	☐	Eukaryotic cell lines
☒	☐	Palaeontology
☒	☐	Animals and other organisms
☐	☒	Human research participants

Methods

n/a	Involved in the study	
☒	☐	ChIP-seq
☐	☒	Flow cytometry
☒	☐	MRI-based neuroimaging

Human research participants

Policy information about studies involving human research participants

Population characteristics	Cord blood was obtained from preterm infants (<33 weeks) and term infants (>38 to <41 weeks) born by elective caesarean section in absence of labor, and peripheral blood was obtained from healthy adult volunteers recruited at the BC Children's Hospital Research Institute. The clinical characteristics of the preterm subjects are detailed in the Supplementary Data 1 file.
Recruitment	Participants were randomly approached for recruitment, sequentially. Women at risk of delivery preterm below 33 weeks were approached during admission to the BC Women's Hospital. Women undergoing an elective Cesarean section >38 weeks at BC Women's Hospital were approached on the morning of delivery, and invited to participate to this research. Healthy adults were randomly approached during normal work hours, for blood donation at the BC Children's Hospital Research Institute.

Flow Cytometry

Plots

Confirm that:

☒ The axis labels state the marker and fluorochrome used (e.g. CD4-FITC).

☒ The axis scales are clearly visible. Include numbers along axes only for bottom left plot of group (a 'group' is an analysis of identical markers).

☒ All plots are contour plots with outliers or pseudocolor plots.

☒ A numerical value for number of cells or percentage (with statistics) is provided.

Methodology

Sample preparation	a BC LSR II flow cytometer (Becton Dickinson)
Instrument	Data were acquired on an BD LSR II flow cytometer (Becton Dickinson)
Software	Data were acquired using the built-in software on the BD LSR II flow cytometry instrument and analyzed using FlowJo v10 (FlowJo, LLC, Ashling OR).
Cell population abundance	Monocyte purity was regularly determined by flow cytometry (FSC/SSC plus staining for CD14 surface expression) throughout the study, and strictly confirmed to be >95% for example, for microarray studies.
Gating strategy	All gating strategies are included in the manuscript where necessary.

☒ Tick this box to confirm that a figure exemplifying the gating strategy is provided in the Supplementary Information.
